# Immunomodulatory effects of probiotic *Lactobacillus brevis* ZG2488 on SARS-CoV-2 vaccine responses in mice

**DOI:** 10.3389/fmicb.2025.1655383

**Published:** 2025-10-14

**Authors:** Mengshan Chen, Zhijie Cao, Yulu Chen, Zhihong Ren, Simin Lu, Danna Pu, Sihui Zhang, Gui Zhang, Jing Yang, Ji Pu, Jianguo Xu

**Affiliations:** ^1^School of Medicine, Nankai University, Tianjin, China; ^2^National Key Laboratory of Intelligent Tracking and Forecasting for Infectious Diseases, National Institute for Communicable Disease Control and Prevention, Beijing, China; ^3^Research Unit for Unknown Microbe, Institute of Pathogen Biology, Chinese Academy of Medical Sciences and Peking Union Medical College, Beijing, China; ^4^Department of Epidemiology and Biostatistics, School of Public Health, Peking University, Beijing, China; ^5^Infection Management Office, Shandong Provincial Hospital Affiliated to Shandong First Medical University, Jinan, China; ^6^Research Center for Reverse Microbial Etiology, Workstation of Academician, Shanxi Medical University, Taiyuan, China

**Keywords:** *Lactobacillus brevis*, SARS-CoV-2 vaccine, probiotics, transcriptomic analysis, immune cell reshaping

## Abstract

**Introduction:**

This study investigates the immunoenhancing effects of the probiotic *Lactobacillus brevis* ZG2488 on an adenovirus-vectored SARS-CoV-2 vaccine (AdC68-Delta-S) in mice.

**Methods:**

Mice were administered ZG2488 in combination with AdC68-Delta-S. Immune responses were evaluated by measuring SARS-CoV-2-specific IgG in bronchoalveolar lavage fluid (BALF), IFN-γ secretion by splenocytes, and transcriptomic and CIBERSORT analyses of splenic immune cells.

**Results:**

ZG2488 intervention significantly enhanced local mucosal humoral immunity in the respiratory tract (increased SARS-CoV-2-specific IgG in BALF) and systemic Th1 cellular immunity (increased IFN-γ secretion by splenocytes). Transcriptomic analysis revealed upregulation of the JAK-STAT signaling pathway, antigen processing and presentation pathways, and pro-inflammatory pathways (IL-17/TNF/NLR/PI3K-Akt), alongside downregulation of hyperinflammation-associated pathways. CIBERSORT analysis showed that ZG2488 reshaped splenic immune cell composition, increasing memory CD4^+^ T cells, Th1 cells, and dendritic cells (DCs), while decreasing macrophages, follicular helper T (Tfh) cells, monocytes, and γδ T cells.

**Discussion:**

These findings demonstrate that L. brevis ZG2488 enhances the immune response to the SARS-CoV-2 vaccine through synergistic and multi-mechanistic actions, supporting its potential as a probiotic adjuvant strategy.

## 1 Introduction

With the escalating threat of viral infections, the development of effective vaccine strategies has become critically important (Piot et al., 2019). Although a variety of vaccines are already in widespread use, their immunogenicity remains suboptimal, particularly in immunocompromised or high-risk populations (Meng et al., 2022). Therefore, improving vaccine efficacy, especially in these high-risk groups, is of paramount importance.

In recent years, research has shown that probiotics, when used as immunoadjuvants, can enhance vaccine-induced immune responses by modulating both gut and systemic immunity (Lin et al., 2021; Peroni and Morelli, 2021). Probiotics from the *Lactobacillus* genus, including *Lactobacillus brevis*, have been demonstrated to regulate immune responses through mechanisms such as cytokine production (Yin et al., 2023; Zielińska et al., 2019), T cell responses (Karimi et al., 2009; Maassen et al., 2000), and antigen presentation (Prado Acosta et al., 2021), thereby enhancing immune protection.

*Lactobacillus brevis* ZG2488 (hereafter referred to as *L. brevis* ZG2488) is a novel strain isolated by our research group from the feces of healthy humans (Cao et al., 2025). In preliminary studies, we found that ZG2488 exhibits significant probiotic characteristics, including resistance to artificial gastric juice and bile salts, high adhesion ability, and antimicrobial activity. Further research has shown that ZG2488 can enhance nitric oxide production and modulate the expression of pro-inflammatory cytokines (such as IL-1β, IL-6, and TNF-α) in macrophages, thereby positively influencing immune responses (Cao et al., 2025). Despite its demonstrated immunomodulatory effects, the role of ZG2488 in enhancing the immunogenicity of the SARS-CoV-2 vaccine remains understudied.

This study hypothesizes that *Lactobacillus brevis* ZG2488, through its immunomodulatory properties, can enhance the immune response to the SARS-CoV-2 vaccine, particularly in mucosal and cellular immunity, thus improving the overall vaccine efficacy. To test this hypothesis, we assessed the effects of ZG2488 on the immune response in a mouse model, specifically evaluating its ability to enhance the immunogenicity of the SARS-CoV-2 vaccine (AdC68-Delta-S). We focused on examining the impact of ZG2488 on local mucosal immune responses, systemic cellular immunity, and immune cell infiltration patterns, and further explored its potential molecular mechanisms through transcriptomic analysis.

## 2 Methods

### 2.1 Bacterial culture

*Lactobacillus brevis* ZG2488 isolated from healthy human feces was grown in De Man Rogosa and Sharpe (MRS) agar plate at 37 °C for 24 h under aerobic condition. The logarithmic phase of *L. brevis* ZG2488 was washed and resuspended in sterile phosphate-buffered saline (PBS) for oral inoculation of mice. *Lactobacillus plantarum* GUANKE (LPG) was used as a comparative strain owing to its boosting immune response to the vaccine (Xu et al., 2021). It was also isolated from the feces of healthy people and grown in MRS plate at 37 °C for 24 h under aerobic condition.

### 2.2 Animal experimental design

**Figure 1:**
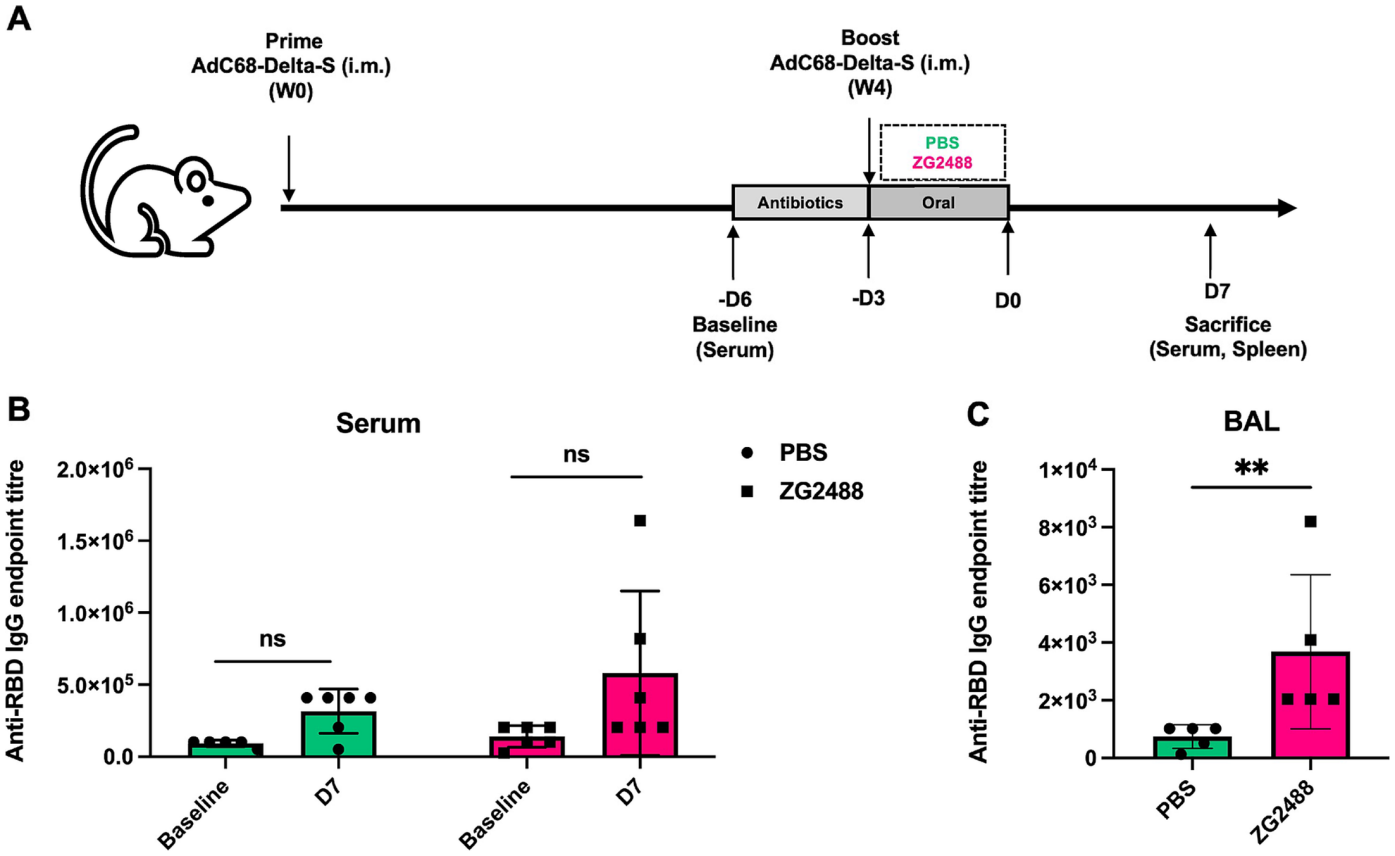
Effect of *Lactobacillus brevis* ZG2488 on SARS-CoV-2-specific IgG levels in serum and BALF of mice following AdC68-Delta-S vaccination. **(A)** Schematic overview of experimental design. **(B)** The concentration of anti-RBD IgG endpoint titer in serum at baseline and day 7 (D7) post-vaccination in PBS and ZG2488-treated mice. No significant difference was observed between groups. **(C)** Anti-RBD IgG endpoint titer in BALF at D7 post-vaccination. ZG2488 treatment significantly increased IgG levels compared to PBS (*p* < 0.01). Data are presented as median ± interquartile range. ns, not significant, ^**^p < 0.01.

Institute of Cancer Research (ICR) mice (*n* = 5 per group) were primed and boosted via intramuscular injection with AdC68-Delta-S vaccine (5 × 10^10^ viral particles per dose) at weeks 0 and 4 (Xu et al., 2021). Beginning 3 days prior to the booster immunization, mice were provided with drinking water containing 1 g/L of ampicillin daily to deplete gut microbiota. Subsequently, mice were orally gavaged with ZG2488 (5 × 10^9^ CFU/200 μL) or phosphate-buffered saline (PBS; control group) for 3 consecutive days; 7 days post-intervention, mice were sacrificed, and serum, bronchoalveolar lavage fluid (BALF), and spleens were collected for analysis. Schematic overview of experimental design is shown in Figure 1A.

### 2.3 Ethics statement and animals

Specific pathogen-free (SPF) female ICR mice (6–8 weeks old) were procured from the Beijing Vital River Laboratory Animal Technology Co., Ltd. [Laboratory Animal Permit No. SYXK (Jing) 2022-0029] and housed in the Laboratory Animal Center of the Chinese Center for Disease Control and Prevention. All experimental procedures involving animals were approved by the Institutional Animal Care and Use Committee (IACUC) of the same center (Approval Code: 2022-023) and conducted in accordance with national and institutional guidelines for animal care and use.

### 2.4 ELISpot assay

Splenocytes were isolated from vaccinated mice and resuspended in complete RPMI-1640 medium. Cells were seeded at 5 × 10^5^ cells per well in 96-well ELISpot plates pre-coated with anti-mouse IFN-γ capture antibody. The splenocytes were stimulated with RBD peptide pool (15-mer peptides overlapping by 11 amino acids, spanning the SARS-CoV-2 Delta variant spike RBD domain) at 2 μg/mL per peptide (Xu et al., 2021). Positive control wells received PMA (50 ng/mL) plus ionomycin (1 μg/mL), while negative control wells contained medium only. After 20 h in a 5% CO_2_ atmosphere, cells were removed and plates were processed using the mouse IFN-γ ELISpot kit (BD Bioscience, #551083) following manufacturer’s instructions: biotinylated detection antibody incubation (2 h, room temperature), streptavidin-HRP conjugate incubation (1 h, room temperature), and development with AEC substrate (15 min). IFN-γ-producing spot-forming cells (SFCs) were quantified using an iSpot Spectrum plate reader (AID Autoimmun Diagnostika GmbH). Results were expressed as IFN-γ SFCs per 10^6^ splenocytes after subtracting negative control values.

### 2.5 Antibody detection

RBD-specific antibody titers in serum and BAL fluid were quantified via ELISA. Samples were heat-inactivated (56 °C for 30 min) prior to analysis. 96-well plates were coated with 100 μL/well recombinant SARS-CoV-2 RBD protein (1 mg/mL; Z03483-1, Genscript) overnight at 4 °C. After PBS-T (0.5% Tween-20) washes, plates were blocked with PBS-T/5% non-fat milk (200 μL/well, 2 h, RT). Serial two-fold dilutions (initial 1:100) of samples were added and incubated (3 h, RT). For IgG detection, HRP-conjugated goat anti-mouse IgG (1:5,000; ZB-5305, Zsbio) in PBS-T/5% milk was added (100 μL/well, 1 h, RT). Following PBS-T washes, OPD substrate (SIGMAFAST OPD tablet SLCC0308 in 20 mL H_2_O) was added. Reaction was stopped with 1 M H_2_SO_4_ after 5 min (RT), and absorbance was read at 492 nm (Synergy Microplate Reader, Bio-Tek). Endpoint titers were defined as the highest dilution with OD_492_ value greater than twice the background signal.

### 2.6 Transcriptomic and immune cell infiltration analysis

RNA sequencing was performed on splenocytes using the Illumina NovaSeq 6000 platform (Illumina, San Diego, CA). RNA libraries were prepared using the TruSeq Stranded mRNA Library Prep Kit (Illumina, San Diego, CA) according to the manufacturer’s instructions. Differentially expressed genes (DEGs) were analyzed using DESeq2 (version 1.30.0), and gene set enrichment analysis (GSEA) was performed with the GSEA software (version 4.1.0). The proportions of splenic immune cell subsets were inferred using the CIBERSORT (version 1.03) deconvolution algorithm. Statistical analysis was performed using GraphPad Prism (version 9.0.0), with *p* < 0.05 considered statistically significant.

### 2.7 Data analysis

Statistical analyses were conducted using GraphPad Prism version 10.0 (GraphPad Software, Inc., San Diego, CA, United States). Experimental values are expressed as mean ± standard deviation (SD). Intergroup differences were evaluated as follows: comparisons between two groups utilized two-tailed unpaired Student’s *t*-tests with Welch’s correction. For comparisons involving three or more groups, one-way analysis of variance (ANOVA) was applied, followed by Dunnett’s *post hoc* test for multiple comparisons. A *p*-value <0.05 was considered statistically significant, with significance levels denoted as ^*^p < 0.05, ^**^p < 0.01, and ^***^p < 0.001. For transcriptome data analysis, the Benjamini–Hochberg method was used to perform false discovery rate (FDR) correction on the *p*-values obtained from DESeq2 and GSEA analyses. All reported *p*-values for differentially expressed genes and enriched pathways are FDR-adjusted values, with the significance threshold set at FDR <0.1.

## 3 Results

### 3.1 *Lactobacillus brevis* ZG2488 enhances SARS-CoV-2 IgG antibody against SARS-CoV-2 in the respiratory tract

We first evaluated the levels of SARS-CoV-2-specific IgG antibodies in serum and bronchoalveolar lavage fluid (BALF). Results showed that the level of SARS-CoV-2-specific IgG antibodies in BALF was significantly higher in the *L. brevis* ZG2488 intervention group compared to the PBS control group (*p* < 0.05, Figure 1B). However, although the serum SARS-CoV-2 IgG antibody level showed an upward trend in the probiotic group, the difference between the two groups did not reach statistical significance (*p* > 0.05, Figure 1C). These results indicate that *L. brevis* ZG2488 preferentially enhances the respiratory tract’s local mucosal immune response, significantly promoting the production of SARS-CoV-2-specific antibodies in BALF, suggesting an important role for probiotics in local immunomodulation.

### 3.2 *Lactobacillus brevis* ZG2488 enhances antigen-specific Th1 cellular immune responses both *in vivo* and *in vitro*

**Figure 2:**
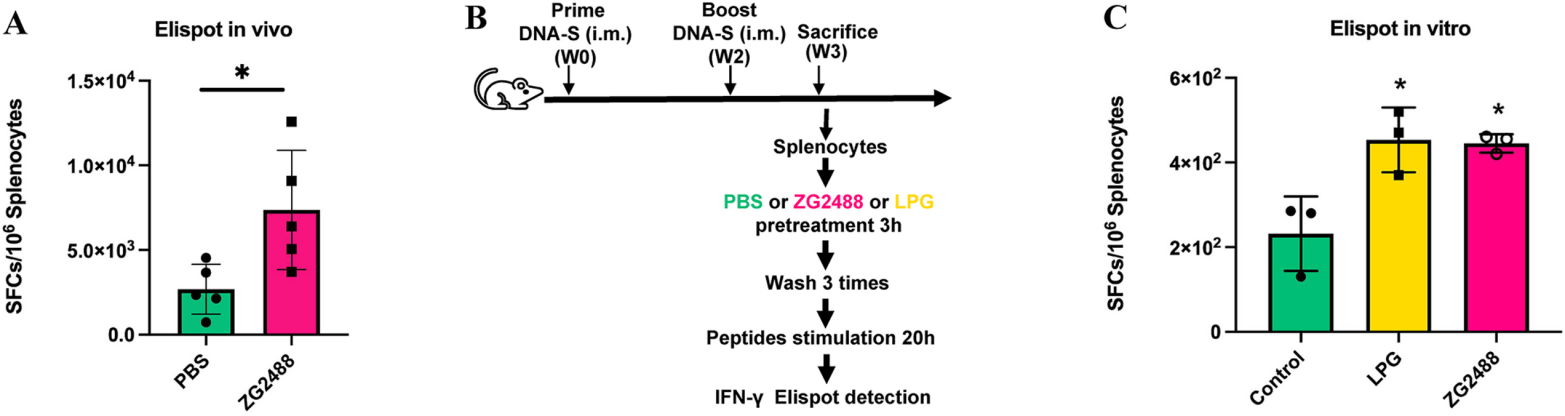
*Lactobacillus brevis* ZG2488 enhances Th1-type immune response to the AdC68-Delta-S vaccine. **(A)** *In vivo* ELISpot analysis showing increased IFN-γ producing splenocytes in the ZG2488-treated group compared to PBS controls (*p* < 0.05). **(B)** ELISpot analysis of splenocytes from *in vitro* culture with LPG, ZG2488, and PBS, showing significantly higher IFN-γ production in the ZG2488 group (*p* < 0.05). **(C)** Comparison with the positive control, LGP, confirmed ZG2488’s ability to stimulate IFN-γ production *in vitro*. Data are shown as mean ± SEM, ^*^p < 0.05.

To evaluate the effect of *L. brevis* ZG2488 on systemic cellular immunity *in vivo*, splenocytes were isolated from mice administered *L. brevis* ZG2488 or PBS after vaccination and subjected to an ELISpot assay. Compared to the PBS control group, the number of IFN-γ-producing spot-forming cells (SFCs) in the spleens of the *L. brevis* ZG2488 group was significantly increased (*p* < 0.05, Figure 2A), clearly indicating that *in vivo L. brevis* ZG2488 effectively enhanced the antigen-specific IFN-γ response of splenocytes to the SARS-CoV-2 vaccine, suggesting enhanced Th1-type cellular immunity.

To assess the direct immunomodulatory effect of *L. brevis* ZG2488, splenocytes were harvested from vaccinated, non-probiotic-treated healthy mice, co-cultured *in vitro* with probiotics or PBS, and analyzed by ELISpot assay. Results found that compared to the PBS co-culture group, probiotic co-culture significantly increased the number of IFN-γ-producing SFCs in splenocytes (*p* < 0.05, Figures 2B,C), and the level of IFN-γ SFCs induced by *L. brevis* ZG2488 was not significantly different from that of the positive control strain co-culture group [*L. plantarum* GUANKE, previously confirmed by our laboratory to enhance IFN-γ production capability (Xu et al., 2021)] (*p* > 0.05). This *in vitro* result directly confirms that *L. brevis* ZG2488 possesses the immunomodulatory activity to directly stimulate splenocytes and promote IFN-γ production, with a stimulatory potency equivalent to that of the known effective positive control strain. *In vivo* intervention and *in vitro* co-culture experiments consistently demonstrate that *L. brevis* ZG2488 effectively promotes IFN-γ production by splenocytes in response to the SARS-CoV-2 vaccine antigen, strongly supporting the role of *L. brevis* ZG2488 in optimizing vaccine efficacy by enhancing Th1-type cellular immune responses.

### 3.3 *Lactobacillus brevis* ZG2488 specifically augments the splenocyte IFN-γ signaling axis

**Figure 3:**
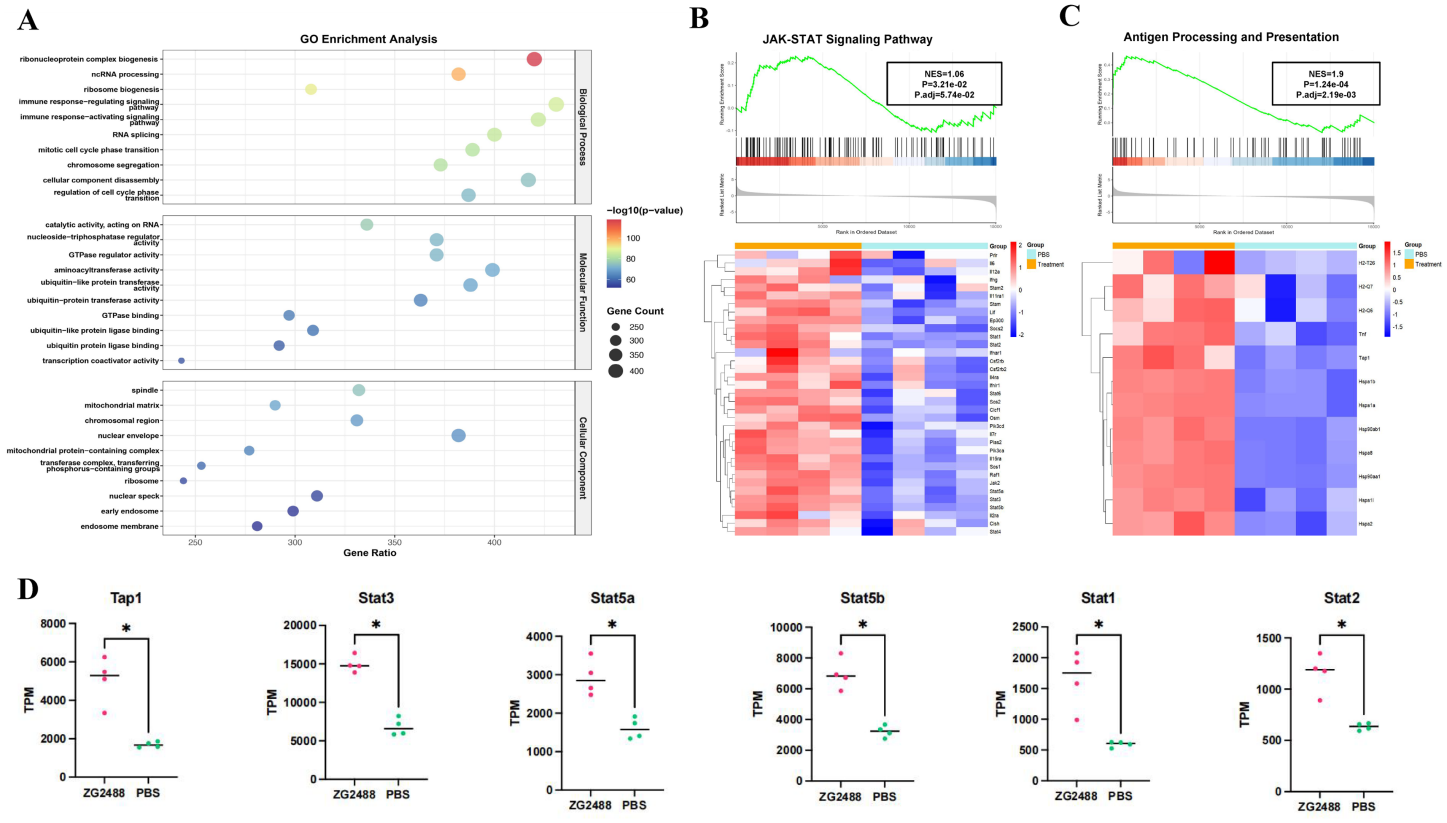
Transcriptomic analysis reveals activation of the JAK–STAT signaling pathway and antigen processing in ZG2488-treated mice. **(A)** GO enrichment analysis. **(B,C)** Gene set enrichment analysis (GSEA) of the JAK–STAT pathway and antigen processing and presentation pathway showing significant upregulations in ZG2488-treated mice. The heatmaps show the expression levels of key genes in these pathways. Statistical significance was determined using FDR adjusted *p*-values (Benjamini–Hochberg correction). **(D)** Expression levels of selected genes involved in JAK–STAT pathway in ZG2488-treated and PBS-treated groups. Data are presented as mean ± SD. ^*^p < 0.05 and ^**^p < 0.01.

Transcriptomic analysis of splenocytes indicated that the immune regulatory effects of *L. brevis* ZG2488 intervention were significant. In the GO enrichment analysis, immune response-activating and regulating signaling pathways (such as “immune response-activating signaling pathway” and “immune response-regulating signaling pathway”) are significantly enriched, indicating the involvement of genes in key immune regulation processes related to the SARS-CoV-2 immune response. Additionally, the enrichment of terms related to transcription factors and immune cell activation in “cell cycle” and “immune response” processes suggests the immunoenhancing effect of the COVID-19 vaccine (Figure 3A).

GSEA enrichment analysis further elucidated the mechanistic basis for the enhanced immune response, specifically revealing the activation of core pathways involved in IFN-γ production and signaling following *L. brevis* ZG2488 intervention. First, the JAK–STAT signaling pathway was significantly enriched and upregulated in the *L. brevis* ZG2488 group (FDR < 0.05, normalized enrichment score NES = 1.06, Figure 3B), with increased expression of key genes (such as *Ifng*, *Stat1*, *Stat2*, *Jak2*, and Supplementary Figure S1 and Figure 3D). Since JAK–STAT is the core downstream cascade of IFN-γ receptor signaling (He et al., 2023; Lee and Benveniste, 1996; Xu et al., 2021), this upregulation provides the mechanistic basis for the enhanced IFN-γ secretion observed in the ELISpot assay (Figures 2A,C). Second, genes related to antigen processing and presentation were coordinately upregulated (FDR < 0.01, NES = 1.9, Figure 3C), involving increased expression of MHC-I genes (*Tap1*), MHC-II genes (*H2-T26*, *H2-Q7*, and *H2-Q6*), and antigen presentation-related genes (*Hsp90ab1*, *Hsp90aa1*, *Hsp1b*, and *Hsp1a*), indicating enhanced antigen-presenting cell (APC) function (Tesniere et al., 2008). This is consistent with the results of enhanced antigen-specific T cell activation and potent IFN-γ responses to the vaccine antigen (Figure 2A). These results demonstrate that *L. brevis* ZG2488 directly reinforces Th1-polarized cellular immunity by transcriptionally coordinating enhanced antigen presentation efficiency and amplification of the IFN-γ–JAK–STAT signaling program. The upregulation of IFN-γ, JAK–STAT pathway components (*Stat1*, *Stat2*, *Jak2*), and MHC-I/II genes, all key markers of Th1 immune responses (He et al., 2023), indicates the promotion of Th1 polarization, a hallmark of cellular immunity against intracellular pathogens. Although qPCR validation for key transcriptional changes was not performed due to sample limitations, the RNA-seq findings for these core pathway genes were supported by stringent statistical correction and, most importantly, are highly consistent with the observed functional immune outcomes.

### 3.4 *Lactobacillus brevis* ZG2488 pleiotropically modulates accessory immune and inflammatory pathways

**Figure 4:**
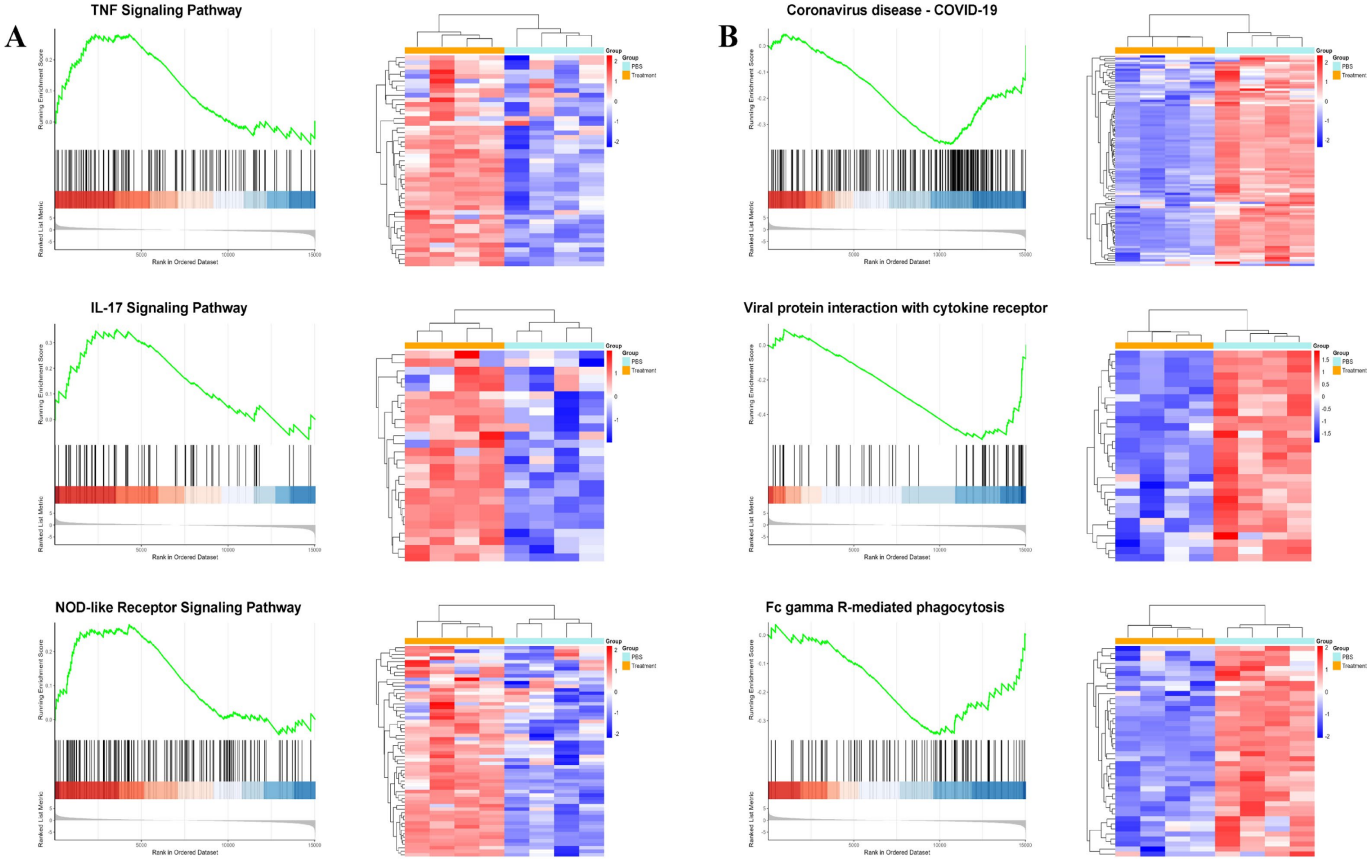
*Lactobacillus brevis* ZG2488 modulates immune and inflammatory pathways. **(A)** Upregulation of pro-inflammatory pathways, including IL-17, TNF, and NOD-like receptor signaling in ZG2488-treated mice. **(B)** Downregulation of immune-related pathways, such as viral protein-cytokine receptor interaction and coronavirus disease pathways. GSEA results for these pathways are shown with NES scores and adjusted *p*-values. Statistical significance was determined using FDR adjusted *p*-values (Benjamini–Hochberg correction). Data indicate modulation of inflammatory and antiviral responses. ^*^p < 0.05 and ^**^p < 0.01.

Beyond the core IFN-γ axis, *L. brevis* ZG2488 differentially regulated multiple immune networks, reflecting its pleiotropic immunomodulatory effects. First, pro-inflammatory and innate immune pathways such as IL-17 signaling (FDR <0.05, NES = 1.446), TNF signaling (FDR <0.01, NES = 1.9), and NOD-like receptor signaling (FDR <0.05, NES = 1.31) were upregulated (Figure 4A), indicating synchronized enhancement of innate immune recognition, inflammatory responses, and cell survival signals.

Second, genes expression in pathways such as viral protein-cytokine receptor interaction (FDR <0.05, NES = −1.927), coronavirus disease-COVID-19 (FDR <0.01, NES = −1.564), and FcγR-mediated phagocytosis (FDR <0.05, NES = −1.319) was suppressed (Figure 4B). This selective downregulation may prevent excessive inflammation or resource exhaustion during the immune priming phase induced by vaccination. *L. brevis* ZG2488 shapes a balanced immunotranscriptomic landscape, enhancing protective Th1 and innate immunity while actively suppressing hyperinflammation and virus susceptibility-related pathways.

### 3.5 *Lactobacillus brevis* ZG2488 alters the immune cell infiltration landscape in the spleen

**Figure 5:**
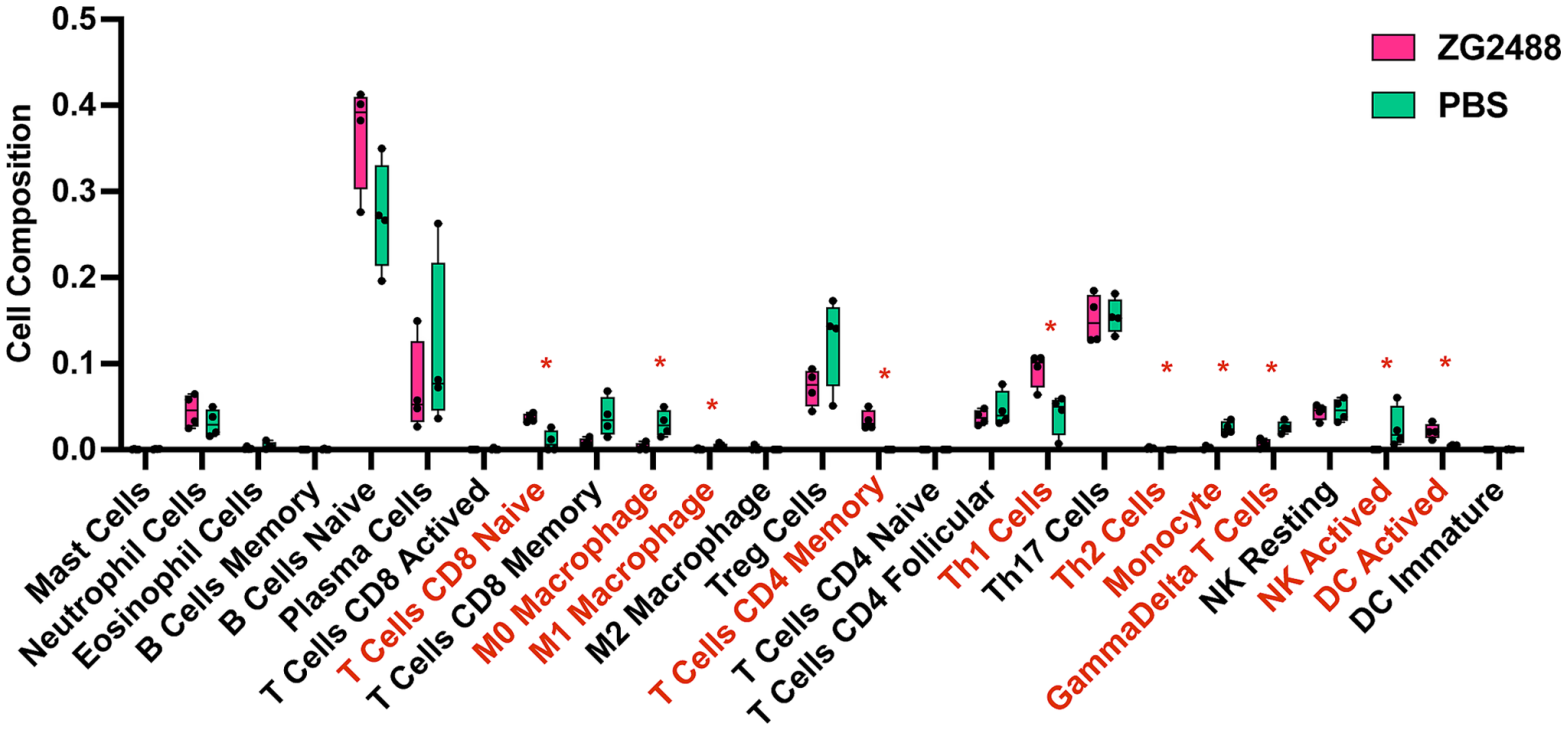
*Lactobacillus brevis* ZG2488 alters immune cell infiltration in the spleen. CIBERSORT analysis shows significant changes in the immune cell composition of spleens from ZG2488-treated versus PBS-treated mice. The ZG2488 treatment increased the proportions of memory CD4^+^ T cells, Th1 cells, and dendritic cells, while decreasing macrophages, monocytes, and γδ T cells. Data are shown as boxplots overlaid with individual data points. TPM, transcripts per million. Asterisks indicate significant differences. ^*^p < 0.05.

According to CIBERSORT analysis results (Figure 5), there were significant differences in splenic immune cell composition between the *L. brevis* ZG2488 intervention group and the PBS group. The probiotic group showed higher proportions of memory CD4^+^ T cells, Th1 cells, and dendritic cells (DCs). These results are consistent with the upregulation of antigen processing and presentation-related genes (Figure 3B). Specifically, the increased infiltration of dendritic cells indicates an enhanced role for antigen presentation in the immune response. In contrast, the probiotic intervention group had lower infiltration of macrophages, monocytes, and γδ T cells. These changes in immune cell populations suggest that *L. brevis* ZG2488 may prevent excessive inflammatory responses by suppressing overactive immune cell populations (such as macrophages and monocytes). These alterations in immune cell subsets suggest that probiotic intervention reshapes the immune landscape by enhancing adaptive immune memory (e.g., memory CD4^+^ T cells), promoting Th1 responses, and increasing dendritic cell abundance, while concurrently reducing the proportions of pro-inflammatory cell populations (e.g., macrophages and monocytes). This shift is consistent with an immune profile associated with effective vaccine responses. Overall, the alteration in immune cell infiltration patterns induced by *L. brevis* ZG2488 helps explain the enhanced cellular immunity (e.g., IFN-γ production) and optimized immune response.

## 4 Discussion

This study aimed to investigate the impact of *L. brevis* ZG2488 on the immune response to the SARS-CoV-2 vaccine in mice, focusing on various immune parameters such as SARS-CoV-2-specific IgG antibodies, IFN-γ secretion, immune cell infiltration, and transcriptomic changes. Our results indicate that *L. brevis* ZG2488 significantly enhanced antigen-specific antibody and T-cell responses, providing evidence for its immunomodulatory potential in improving vaccine efficacy (Redenti et al., 2024; Ryan et al., 2025; Zhu et al., 2025). Beyond mechanistic insights, our findings suggest the potential clinical relevance of ZG2488 as a candidate vaccine adjuvant. Orally administered ZG2488 was associated with enhanced mucosal humoral immunity (elevated BALF IgG) and systemic Th1-cell-mediated immunity (increased IFN-γ production). This dual activity may be advantageous against respiratory pathogens such as SARS-CoV-2, where immunity at the mucosal site of entry could contribute to overall protection. Further clinical studies are needed to evaluate the translational potential of ZG2488-adjuvanted vaccination in humans.

*Lactobacillus brevis* ZG2488 preferentially enhanced local mucosal immunity in the respiratory tract, as evidenced by a significant increase in SARS-CoV-2-specific IgG antibodies in bronchoalveolar lavage fluid (BALF), while serum IgG showed an upward trend but did not reach statistical significance. This result is consistent with previous studies suggesting that probiotics not only modulate systemic immunity but also enhance local immune responses, particularly in mucosal tissues (Jin et al., 2021; Ma et al., 2023; Tseng et al., 2025; Zhao et al., 2024). *Lactobacilli*, especially, are crucial in enhancing local immunity by promoting gut mucosal immune responses and boosting the efficacy of antigen-presenting cells, which are key for improving local vaccine immune responses (Rawling et al., 2023; van Baarlen et al., 2013; Zhang et al., 2023).

In addition to enhancing humoral immunity, *L. brevis* ZG2488 significantly improved the ability of splenocytes to produce IFN-γ in response to the SARS-CoV-2 vaccine, both *in vivo* and *in vitro*. IFN-γ, as a key effector cytokine in the Th1 immune response, is essential for antiviral defense and cellular immunity (Assaf et al., 2017; Lei et al., 2024; Rosloniec et al., 2002). The upregulation of IFN-γ production by *L. brevis* ZG2488 suggests that the probiotic enhances the ability to clear the virus, potentially improving the immune response to SARS-CoV-2. Transcriptomic analysis further revealed the molecular basis of this enhancement, highlighting the upregulation of genes involved in the JAK–STAT signaling pathway (*Ifng*, *Stat1*, *Stat2*, *Jak2*) and the antigen processing and presentation pathway (Tap1, MHC-II genes, Hsps). These findings align with other studies on *Lactobacilli*’s effects on immune regulation, confirming their role in enhancing immune function through activation of key immune signaling pathways (Ding et al., 2017; Ji et al., 2025). We believe that the existing data provides strong support for our mechanistic conclusions. However, due to current limitations in experimental conditions and resources, we were unable to conduct the relevant knockout validation experiments. Should the opportunity arise in the future, we would be eager to consider performing these knockout validations to further strengthen our conclusions.

Moreover, *L. brevis* ZG2488 displayed pleiotropic effects on innate immune signaling pathways, upregulating pro-inflammatory pathways such as IL-17, TNF, NOD-like receptor, and PI3K-Akt. Additionally, this study uncovered the pleiotropic effects of *L. brevis* ZG2488 on several immune signaling pathways. Interestingly, in contrast to earlier studies that found *L. brevis* ZG2488 downregulates TNF-α in macrophages, the current study observed an upregulation of the TNF pathway in splenocytes (Cao et al., 2025). Beyond the enrichment of the TNF signaling pathway by GSEA, we confirmed that the gene encoding the key ligand TNF-α itself was significantly upregulated in the spleens of ZG2488-treated mice compared to PBS controls (Supplementary Figure S2, *p* < 0.05 by *t*-test). This direct evidence at the gene expression level solidifies the conclusion that the TNF pathway activation is a hallmark of the immune response potentiated by *L. brevis* ZG2488. This apparent contradiction may be due to differences in the immune cell types involved and the specific context of the immune environment (Adami et al., 2020). While the downregulation of TNF-α in macrophages likely represents a protective effect against excessive innate inflammation, the upregulation of the TNF signaling pathway in vaccine-primed splenocytes suggests a potentiation of adaptive immunity. Many studies have demonstrated the crucial role of TNF-α in the early stages of vaccination (Maggioli et al., 2016). For example, research has shown that individuals receiving TNF inhibitors have a reduced response to the flu vaccine (Munusamy Ponnan et al., 2019). Furthermore, after COVID-19 vaccination, this pathway promotes local immune responses, helping to enhance antigen presentation, particularly in antigen processing and T cell activation (Kawakita et al., 2025). This is very important for the early immune response, aiding in the initiation of the immune response and the establishment of immune memory (Procaccini et al., 2021). Therefore, the upregulation of the TNF signaling pathway by *L. brevis* ZG2488 in this specific context is not necessarily contradictory but rather highlights its context-dependent immunomodulatory function. It may enhance vaccine responses under conditions that require robust T-cell activation—key processes for a strong cellular immune response (Geisen et al., 2022). This raises the possibility that *L. brevis* ZG2488 may act as an immunoadjuvant by upregulating TNF-α, thus stimulating a more robust inflammatory response, which in turn promotes effective antigen presentation and T-cell activation. This mechanism provides new insights into the potential of *L. brevis* ZG2488 as an adjuvant to enhance vaccine-induced immunity. Notably, while some probiotic strains modulate inflammation by downregulating TNF-α to prevent excessive immune activation, *L. brevis* ZG2488 may function differently by upregulating TNF-α to drive stronger immune responses, particularly in the early stages of immunity. These contrasting effects on TNF-α signaling reflect the strain-specific and context-dependent nature of probiotic-mediated immune modulation.

Similarly, IL-17 signaling contributes to mucosal immunity and defense against extracellular pathogens. Although dysregulated IL-17 can drive autoimmunity, its induction following vaccination supports neutrophil recruitment, barrier integrity, and antibody-mediated protection. In the context of a respiratory-targeted vaccine, this Th17-skewed response may enhance mucosal immunity in the airways (Schnoeller et al., 2014; Wu et al., 2021). The NOD-like receptor signaling pathway is involved in inflammasome activation and innate immune sensing. While aberrant NLR signaling can cause excessive inflammation, its role in vaccine adjuvancy is well-established. NLR activation promotes cytokine production and co-stimulatory molecule expression, thereby bridging innate and adaptive immunity (Higgins and Mills, 2010; Maisonneuve et al., 2014). Moreover, the PI3K-Akt signaling pathway, crucial for cell proliferation, survival, and immune responses, was also upregulated (Glaviano et al., 2023; Manning and Cantley, 2007). By activating this pathway, *L. brevis* ZG2488 may enhance immune cell proliferation, particularly T cells and B cells, further promoting a stronger immune defense. Importantly, the potential for immunopathology appears to be balanced by the concurrent downregulation of hyperinflammation-related pathways, such as viral protein-cytokine interactions and FcγR-mediated phagocytosis, as shown in our GSEA results (Figure 4B). This coordinated regulation—enhancing vaccine-relevant inflammation while suppressing nonspecific hyperactivation—suggests that ZG2488 promotes a focused and adaptive immune response rather than indiscriminate inflammation. Thus, the upregulation of these pathways is likely a hallmark of effective immune stimulation rather than uncontrolled inflammation. Future studies involving cytokine blockade or challenge models could further confirm the functional balance of these responses.

Conversely, *L. brevis* ZG2488 also selectively downregulated pathways like viral protein-cytokine receptor interaction, coronavirus disease-COVID-19, and FcγR-mediated phagocytosis. This selective downregulation could prevent excessive inflammation or resource depletion during the immune priming phase, helping to maintain a balanced immune response. These findings illustrate the complex and bidirectional nature of probiotic-mediated immune modulation, where probiotics like *L. brevis* ZG2488 may enhance immune responses while preventing potential harmful overactivation of certain immune pathways.

CIBERSORT analysis revealed that *L. brevis* ZG2488 modulates immune cell infiltration in the spleen, increasing the proportion of memory CD4^+^ T cells, Th1 cells, and DCs, while decreasing macrophages, CD4^+^ Tfh cells, monocytes, and γδ T cells. The increased DCs correlate with upregulated antigen presentation genes, which support the initiation of adaptive immune responses (Hilligan and Ronchese, 2020). The increased presence of memory CD4^+^ T and Th1 cells is indicative of a strengthened Th1 response, suggesting potential for long-term protection. The reduction in macrophages, monocytes, and Tfh cells may reflect an optimization of the immune response, suppressing overactive or potentially pro-inflammatory cell populations, thereby preventing excessive inflammation or adverse immune reactions. *Lactobacilli* are known to modulate immune cell infiltration in similar ways, enhancing the activity of memory T cells and Th1 cells while suppressing the overactivation of macrophages and γδ T cells to avoid excessive immune activation (Finamore et al., 2019; Liu et al., 2014; Min et al., 2023).

In summary, *Lactobacillus brevis* ZG2488 significantly enhanced the immune response to the SARS-CoV-2 vaccine in mice. This effect is achieved through several mechanisms: enhancing antigen presentation, modulating immune cell activity, promoting the secretion of protective cytokines (particularly IFN-γ), regulating immune signaling networks, and reshaping immune cell composition (e.g., increasing protective cells like DCs, Th1 cells, and memory CD4^+^ T cells). These findings underscore the important role of probiotics in enhancing vaccine-induced immunity, particularly through their immunomodulatory effects. We would like to emphasize that this study focused on the peak effector phase at 7 days post-boost to optimally capture the immunomodulatory effects of the probiotic, while future investigations incorporating additional timepoints will be essential to delineate the kinetics of immune response initiation, peak, and memory formation following ZG2488-adjuvanted vaccination.

We acknowledge that the sample size (*n* = 5 per group) is a limitation, although it meets the standard for preliminary mouse studies. However, the consistent and robust effects observed across bodily fluid, cellular, and transcriptome analyses, coupled with rigorous statistical corrections, strengthen our confidence in the conclusions. Future studies utilizing larger cohorts will be valuable for further validating these findings and exploring more subtle effects. Despite these promising results, one limitation of this study is the absence of a control group that received neither antibiotic nor probiotic treatment but was only vaccinated. Consequently, our findings specifically define the immune-enhancing effects of *L. brevis* ZG2488 within an antibiotic-perturbed microbiome background. It should also be noted that all mice in this study underwent antibiotic pretreatment to facilitate probiotic intervention. While this approach ensured that the observed immunomodulatory effects could be attributed to the action of *L. brevis* ZG2488 under controlled conditions, it prevented us from determining the independent impact of microbiota depletion itself on the vaccine response, as well as the potential influence of the probiotic mediated through gut microbiota. Future studies will include a non-antibiotic-treated control group to comprehensively dissect the respective contributions of the microbiome, antibiotic pretreatment, and probiotic intervention to vaccine-induced immune responses, and to clarify the specific roles of the microbiome and the probiotic in vaccine efficacy. Furthermore, while this study provides a foundational characterization of the novel strain *L. brevis* ZG2488, future research would benefit from direct comparisons with well-established probiotic adjuvants, such as LPG. Our group has previously demonstrated the efficacy of LPG in enhancing vaccine-induced immune responses *in vivo* (Xu et al., 2021). A dedicated comparative study, designed specifically to benchmark the efficacy of ZG2488 against LPG and other documented strains, would offer valuable insights into their relative potency and potential synergistic effects. Such research would significantly advance our understanding of the structure–activity relationships and precise mechanisms of action of different probiotic strains within the immune system. Also, while our study demonstrates enhanced immunity through antibody and IFN-γ ELISpot assays supported by transcriptomic mechanistic insights, it does not fully explore all immune functional outcomes. Future studies should include additional assays such as flow cytometry or viral neutralization to further define *L. brevis* ZG2488’s immunomodulatory scope. Finally, it is important to note that while our transcriptomic and immune deconvolution analyses provide a comprehensive and multi-faceted view of the immunomodulatory effects of *L. brevis* ZG2488, they are inherently correlative and descriptive. The causal mechanisms underlying these observations remain to be formally established. However, the strong coherence between the gene expression signatures, pathway activities, and functional immune outcomes (e.g., IFN-γ production) lends strong support to our model. Future studies employing mechanistic approaches, such as adoptive cell transfer or antibody-mediated neutralization of key cytokines like IFN-γ will be essential to definitively prove causal relationships and elucidate the precise molecular mechanisms by which *L. brevis* ZG2488 enhances vaccine immunity. These considerations will help optimize the therapeutic use of probiotics in vaccine adjuvant strategies.

## 5 Conclusion

This study demonstrates that supplementation with *Lactobacillus brevis* ZG2488 significantly potentiates the immunogenicity of the SARS-CoV-2 AdC68-Delta-S vaccine in mice through multifaceted immunomodulatory mechanisms. Mechanistically, ZG2488 orchestrated a dual enhancement of localized and systemic immunity by (1) augmenting mucosal humoral defenses in the respiratory tract, evidenced by elevated SARS-CoV-2-specific IgG in BALF; (2) amplifying systemic cellular immunity via robust IFN-γ production by splenocytes; (3) globally reprogramming immune signaling networks, with transcriptomic profiling revealing targeted upregulation of the IFN-γ-JAK–STAT axis and antigen presentation pathways, synergistic activation of pro-inflammatory pathways (IL-17, TNF, NLR, PI3K-Akt), and selective downregulation of antiviral/immune exhaustion pathways (viral protein–cytokine interactions, COVID-19-related genes, FcγR-mediated phagocytosis); and (4) remodeling splenic immune architecture through expansion of memory CD4^+^ T cells, Th1 subsets, and dendritic cells, coupled with contraction of macrophages, Tfh cells, monocytes, and γδ T cells. Although ZG2488 shows promise in enhancing vaccine-induced immune responses, this study did not assess its effects on clinically relevant outcomes such as protection from viral challenge, longevity of immune memory, or neutralizing antibody activity. Thus, while ZG2488 exhibits immunomodulatory properties supportive of adjuvant function, future studies are necessary to confirm its clinical utility in improving protective efficacy, sustaining immunological memory, and eliciting potent neutralizing antibody responses.

## Data Availability

The datasets presented in this study are publicly available. This data can be found here: https://doi.org/10.6084/m9.figshare.30252403.

## Supplementary material

SUPPLEMENTARY FIGURE S1 Expression of IFN-γ in response to ZG2488 and PBS treatments. The graph shows the transcript per million (TPM) levels of Ifng in samples treated with ZG2488 (pink) and PBS (green). Data points represent individual samples, and the horizontal bars indicate the mean value for each group. No significant difference (ns) in Ifng expression was observed between the two groups.

SUPPLEMENTARY FIGURE S2 Expression of TNF-*α* in response to ZG2488 and PBS treatments. The graph shows the TPM levels of Ifng in samples treated with ZG2488 (pink) and PBS (green). Data points represent individual samples, and the horizontal bars indicate the mean value for each group. A significant difference in TNF-*α* expression was observed between the two groups (^*^*p* < 0.05).
