# Supplementary figures and images for: Immunomodulatory effects of probiotic Lactobacillus brevis ZG2488 on SARS-CoV-2 vaccine responses in mice
Source: Front Microbiol. 2025 Oct 14;16:1655383. doi: 10.3389/fmicb.2025.1655383 (PMC12558997; doi:10.3389/fmicb.2025.1655383)

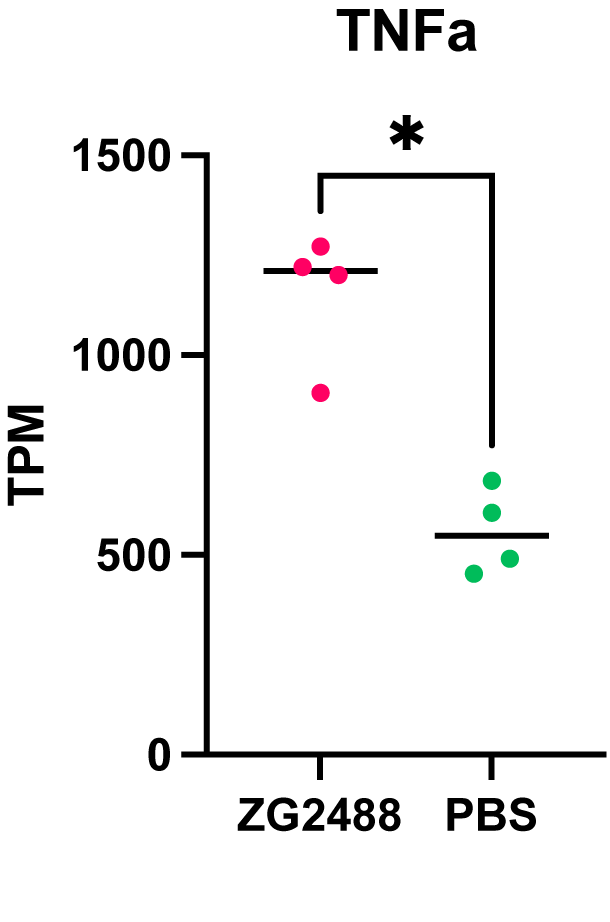

Supplement: SUPPLEMENTARY FIGURE S1 — Expression of IFN-γ in response to ZG2488 and PBS treatments. The graph shows the transcript per million (TPM) levels of Ifng in samples treated with ZG2488 (pink) and PBS (green). Data points represent individual samples, and the horizontal bars indicate the mean value for each group. No significant difference (ns) in Ifng expression was observed between the two groups. [file Image_1.TIF]

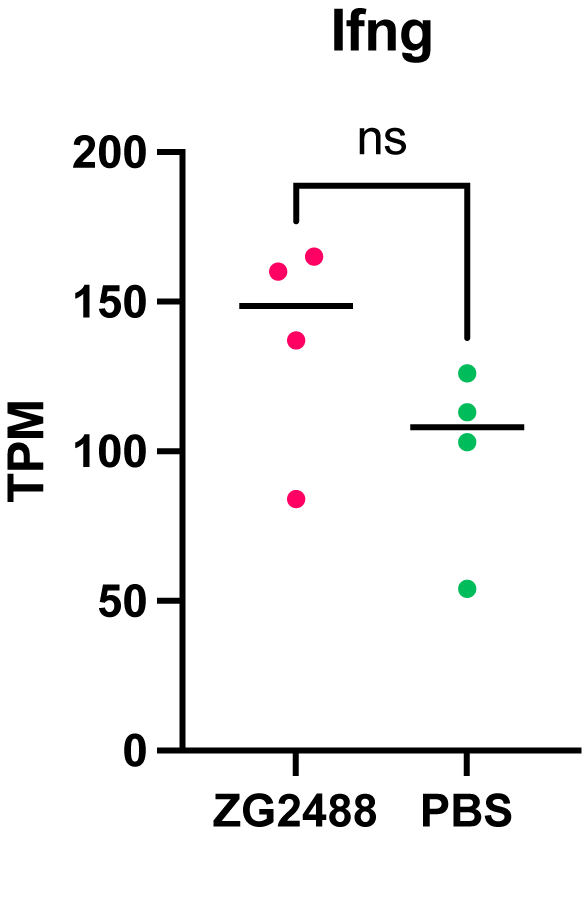

Supplement: SUPPLEMENTARY FIGURE S2 — Expression of TNF-α in response to ZG2488 and PBS treatments. The graph shows the TPM levels of Ifng in samples treated with ZG2488 (pink) and PBS (green). Data points represent individual samples, and the horizontal bars indicate the mean value for each group. A significant difference in TNF-α expression was observed between the two groups (*p < 0.05). [file Image_2.TIF]
